# Assessment of coping: a new french four-factor structure of the brief COPE inventory

**DOI:** 10.1186/s12955-016-0581-9

**Published:** 2017-01-11

**Authors:** Karine Baumstarck, Marine Alessandrini, Zeinab Hamidou, Pascal Auquier, Tanguy Leroy, Laurent Boyer

**Affiliations:** 1 EA 3279, Self-perceived Health Assessment Research Unit, School of Medicine, Aix-Marseille Université, 27 bd Jean Moulin, Marseille cedex 05, Marseille, 13385 France; 2 National Clinical research Quality of Life in Oncology Platform, Marseille, France; 3 Social Psychology Research Group (GRePS EA 4163), Université Lumière Lyon 2, Bron, France

## Abstract

**Background:**

The Brief Coping Orientation to Problems Experienced (Brief COPE) inventory is the most usual measure to identify the nature of coping strategies implemented by individuals and explore 14 coping strategies. The availability of a structure with fewer factors rather than the initial 14-factor structure may be of interest for both healthcare professionals and researchers. We report the validation process of a 4-factor structure of the French version of the Brief COPE in a French sample of individuals facing a singular life event, such as cancer, including patients and their caregivers.

**Methods:**

The cross-sectional study included cancer patients and their caregivers. Self-administered data were collected including: socio-demographic (age, gender, marital status, employment status, and education level), coping strategies using the French version of the Brief COPE, quality of life (QoL) using the French version of the short form health survey questionnaire (SF36). Construct validity, internal consistency, reliability, and external validity were tested.

**Results:**

The sample included 398 individuals. The principal component factor analysis identified a 4-factor structure. The dimensions were labeled according to their constitutive items: social support (8 items), problem solving (4), avoidance (10), and positive thinking (6). The 4-factor structure was supported by different theoretical models of coping and showed satisfactory psychometric properties.

**Conclusion:**

The 4-factor structure of the French version of the Brief COPE, validated in a sample of individuals facing a singular stressful event, including cancer patients and their caregivers, makes the instrument easier to use both in clinical practice and clinical research.

**Electronic supplementary material:**

The online version of this article (doi:10.1186/s12955-016-0581-9) contains supplementary material, which is available to authorized users.

## Background

For different major lifestyle disruptions and stressful situations, there is interest in studying how individuals handle the problems of daily life. These actions, behaviors or thoughts developed to manage stressors are called coping. Coping is commonly defined as the cognitive and behavioral efforts that are implemented to solve problems and reduce the stress that these problems may cause [1, 2]. Several coping strategies can be used in a stressful situation, and the strategies implemented depend both on the individual’s cognitive appraisal of the situation [3, 4] and his/her emotional status. Some authors distinguished emotion-focused and problem-focused strategies [5], whereas others distinguished active and avoidant coping strategies [4]. Standardized measures exist to identify the nature of coping strategies implemented by individuals. The questionnaire the most used in the literature is the Coping Orientation to Problems Experienced (COPE) inventory [6] and its abbreviated version, the Brief COPE [7]. The Brief COPE was designed for ease of administration and a reduced time burden. It is a measure used for many health-relevant situations, such as drug addiction, ageing, breast cancer, depression, and AIDS.

The Brief COPE is based on acknowledged theoretical models, such as Lazarus’ transactional model of stress [2] and the behavioral self-regulation model of Carver and Scheier [6]. It is a multidimensional measure and presents fourteen scales all assessing different coping dimensions. This questionnaire includes 28 items that explore the following 14 coping strategies: self-distraction, active coping, denial, substance use, use of emotional support, use of instrumental support, behavioral disengagement, venting, positive reframing, planning, humor, acceptance, religion, and self-blame. Well-validated language versions of the Brief COPE are currently available [8–10]. The French version of the Brief COPE was validated using standard methods among a large sample of students (*n* = 1834) [11]. French researchers have a relevant tool on hand to measure, as accurately as possible, the coping strategies some people use in everyday life. However, because the inventory assesses 14 different coping strategies, the scores may be hard to synthetize into specific findings for quantitative studies. The availability of a structure with fewer factors, which is easier to use, rather than a 14-factor structure, may be of interest for both healthcare professionals (medical and psychological) and researchers. Four coping profiles may be more manageable and practical for: statistics interpretation, summary of findings, and scientific messages dissemination. Testing this new structure in a population who must face to a stressful event, such as a cancer diagnosis, may be useful to complement the initial cross-cultural validation proposed by Muller [11]. We report the validation process of a 4-factor structure of the French version of the Brief COPE in a French sample of individuals facing a singular life event, such as cancer, including patients and their caregivers.

## Methods

### Design and population

The study used a cross-sectional design. The study sample included ill and (potentially) healthy individuals. The ill individuals were represented by cancer patients with the following inclusion criteria: aged above 18 years; having a cancer defined by histology, loco-regionally advanced or metastatic cancer, with an indication of chemotherapy; able to speak/read French; not having severe cognitive problems based on the physician’s opinion; and agreeing to participate. Healthy individuals were represented by the cancer patients’ caregivers. The main inclusion criteria were as follows: aged above 18 years; designated as the primary caregiver by the patient; able to speak/read French; free from cancer comorbidity; and agreeing to participate. Patients and caregivers were enrolled in two French oncologic departments of public academic teaching hospitals, the Oncology and Therapeutic Innovations Department, Nord Hospital (Marseille) and the Oncology Department, Timone Hospital (Marseille).

### Ethics

Regulatory monitoring was performed in accordance with the French law that requires approval of the French ethics committee (Comité d’éthique, Aix Marseille University, October 8th 2015, Number 2014-09-30-05). Written consent forms for participation were collected from each patient and caregiver.

### Data collection

Self-administered booklets were collected from the patients and caregivers. The booklet included the following data:

- The coping strategies evaluated using the French version of the Brief COPE [7, 11] (dispositional version), an abbreviated version of the COPE inventory [6]. This includes 28 items, scored from one (“I haven’t been doing this at all”) to four (“I’ve been doing this a lot”), exploring 14 strategies: active coping, planning, use of instrumental support, positive reframing, acceptance, use of emotional support, denial, venting, self-blame, humor, religion, self-distraction, substance use and behavioral disengagement. Higher scores reflect a higher tendency to implement the corresponding coping strategies.
- Socio-demographic variables including the following: age, gender, patient-caregiver relationship (partner versus other), marital status (couple versus single), employment status (workers versus non workers), and education level (graduate and university level versus undergraduate).
- Specific information for cancer patients including: cancer location, metastasis, disease duration, and performance status (adopted by the World Health Organization).
- The quality of Life (QoL) assessed using the French version of the short form health survey questionnaire (SF36). It contains 36 items describing 8 dimensions: Physical Functioning (PF); Social Functioning (SF); Role-Physical Problems (RPP); Role-Emotional Problems (REP); Mental Health (MH); Vitality (VIT); Bodily Pain (BP); and General Health (GH). Two composite scores are calculated, the Physical Composite Score (SF36-PCS) and the Mental Composite Score (SF36-MCS). Each dimension is scored within a range from 0 (low QoL level) to 100 (high QoL level) [12, 13].

### Statistical analysis

First, the analysis was conducted on the entire database. Descriptive statistics of the sample included frequencies and percentages of categorical variables and means and standard deviations of continuous variables. The structure of the Brief COPE was studied using an exploratory factor analysis (EFA) performed using maximum-likelihood extraction with varimax rotation on the entire sample. The Kaiser-Meyer-Olkin (KMO) measure of sampling adequacy and Bartlett’s test of sphericity were computed to determine whether the dataset was appropriate for EFA performance [14]. Eigenvalues greater than or equal to one were retained [15]. Items were included in the dimensions if they revealed loading greater than 0.3. In the case of multiple loading of an item on several factors or the case of loading lesser than 0.3, the item was included in the factor that had the most conceptual relationship. The unidimensionality of each dimension was assessed using a Rasch analysis. The goodness-of-fit statistics, the inlier-sensitive fit (INFIT), ranging between 0.7 and 1.3, ensured that all items of the scale measured the same concept [16].

Item internal consistency was assessed by correlating each item with its scale (corrected for overlap) using Pearson’s correlation coefficient (a correlation of 0.4 was recommended for supporting item-internal consistency [17]) . Item discriminant validity was assessed by determining the extent to which items correlated more highly with the dimensions they were hypothesized to represent than with other dimensions [18]. For each dimension scale, the internal consistency reliability was assessed by Cronbach’s alpha coefficient (coefficient of at least 0.7 expected for each scale [17]). Floor and ceiling effects were reported when assessing the homogeneous repartition of the response distribution. Proportions of missing values were provided. The discriminant validity was determined by comparing dimension mean scores of the Brief COPE across individuals groups, such as gender, patient- relationship, living status, employment status, and educational level, and by studying the correlations of Brief COPE scores with age. To explore external validity, relationships between scores of the Brief COPE and scores of SF36 were evaluated. The following hypotheses were formulated: the dimensions of the Brief COPE should differ according to several socio-demographic characteristics (women use external support strategies more often than men, younger people use positive reframing more often than older people, and workers use problem solving more often than non-workers) and ‘active-like’ coping strategies, such as problem solving and positive thinking, should be positively correlated with QoL, whereas ‘passive-like’ coping strategies, such as seeking social support and avoidance, should be negatively correlated with QoL.

Second, the entire database was divided into two sub-samples, the patient sample and the caregiver sample. Confirmatory factor analysis (CFA) based on the LISREL model was separately performed on the two sub-samples to test the adequacy to the predefined model. The adequacy was defined from different indices, the root mean square error of approximation (RMSEA) and comparative fit index (CFI). RMSEA is acceptable if <0.08 and satisfactory if <0.05, and CFI is acceptable if >0.9 [19, 20]. The internal and external validity were explored in the two subsamples following the same procedure described for the entire sample.

Data analyses were performed using SPSS 20.0 computer software, Winsteps for Rasch’s analysis and the structural equation modeling software program Mplus [21] for EFA and CFA.

## Results

### Sample characteristics

A total of 477 individuals were eligible. The sample included 398 individuals with an available Brief COPE. The included individuals (*n* = 398) were significantly older than the 79 non-included and the gender repartition did not differ between the two groups (data not shown). The sample characteristics are presented in Table 1. Patient and caregiver characteristics are available in Additional file 1: Supplementary Table S1.

**Table 1:** Baseline characteristics

		Eligible population	Sample
		*N* = 477	*N* = 398
		*n* (%)	*n* (%)
Gender	Women	263 (55)	221 (56)
	Men	212 (45)	175 (44)
Age (years)	Mean (SD)^a^	59.0 (12.3)	58.2 (13.4)
Sub-samples	Patients	280 (59)	235 (59)
	Caregivers	197 (41)	163 (41)
Educational level	<12 years	244 (52)	196 (50)
	> = 12 years	221 (48)	193 (50)
Living	With a partner	343 (73)	287 (73)
	Alone	129 (27)	107 (27)
Professional status	Workers	106 (23)	90 (23)
	Not workers	366 (77)	303 (77)
Patient-caregiver relationship	Partners	287 (63)	237 (62)
	Not partners	171 (37)	146 (38)

^a^SD standard deviation

### Construct validity

The structure of the Brief COPE was explored using principal component factor analysis, identifying a 4-factor structure accounting for 45% of the total variance (6 to 17% per factor). The dimensions were labeled according to their constitutive items as follows: social support (8 items), problem solving (4), avoidance (10), and positive thinking (6). The structure is presented in Table 2.

**Table 2:** Factor loading of the 28 items of Brief COPE

Items	Factors			
	F1	F2	F3	F4
I’ve been getting comfort and understanding from someone	**0,778**	−0,003	0,102	−0,081
I’ve been getting help and advice from other people	**0,772**	0,192	0,085	0,048
I’ve been saying things to let my unpleasant feelings escape	**0,706**	0,218	0,026	0,200
I’ve been getting emotional support from others	**0,665**	−0,089	0,182	−0,180
I’ve been trying to get advice or help from other people about what to do	**0,642**	0,219	0,113	−0,042
I’ve been expressing my negative feelings	**0,597**	−0,158	0,005	−0,005
I’ve been praying or meditating	**0,326**	0,216	0,425	−0,066
I’ve been trying to find comfort in my religion or spiritual beliefs	**0,318**	0,164	0,431	−0,070
I’ve been taking action to try to make the situation better	0,074	**0,716**	0,035	−0,050
I’ve been concentrating my efforts on doing something about the situation I'm in	0,226	**0,703**	0,030	0,023
I’ve been trying to come up with a strategy about what to do	0,151	**0,651**	0,071	0,073
I’ve been thinking hard about what steps to take	−0,006	**0,619**	0,014	0,185
I’ve been using alcohol or other drugs to help me get through it	−0,095	−0,138	**0,726**	0,288
I’ve been using alcohol or other drugs to make myself feel better	−0,131	−0,165	**0,719**	0,274
I’ve been criticizing myself	0,130	0,109	**0,668**	0,044
I’ve been blaming myself for things that happened	0,128	0,022	**0,608**	−0,146
I’ve been refusing to believe that it has happened	0,239	0,041	**0,474**	−0,395
I’ve been saying to myself “this isn't real”	0,247	0,004	**0,389**	−0,394
I’ve been doing something to think about it less0, such as going to movies0, watching TV…	0,339	−0,035	**0,346**	0,154
I’ve been giving up the attempt to cope	0,154	−0,441	**0,278**	−0,028
I’ve been turning to work or other activities to take my mind off things	0,253	0,191	**0,175**	0,076
I’ve been giving up trying to deal with it	0,266	−0,367	**−0,010**	0,021
I’ve been making jokes about it	0,068	0,037	0,158	**0,762**
I’ve been making fun of the situation	−0,066	−0,008	0,229	**0,634**
I’ve been learning to live with it	0,035	0,334	−0,053	**0,560**
I’ve been accepting the reality of the fact that it has happened	0,041	0,261	−0,235	**0,475**
I’ve been trying to see it in a different light0, to make it seem more positive	0,165	0,539	0,024	**0,456**
I’ve been looking for something good in what is happening	0,119	0,545	0,099	**0,371**

F1 Social support; F2 Problem solving; F3 Avoidance; F4 Positive thinking

### Internal validity

Internal consistency was satisfactory for all dimensions. Each item achieved the 0.40 standard for item-internal consistency, excepted two items of the avoidance factor. The correlation of each item with its contributive dimension was higher than with the others (item discriminant validity). Floor effects ranged from 2.7 to 10.0%, and ceiling effects ranged from 0.3 to 3.3%. Cronbach’s alpha coefficients ranged from 0.71 to 0.82 for three dimensions, indicating satisfactory internal consistency. The overall scalability was satisfactory. No items showed an INFIT statistic outside the acceptable range, except the social support factor. All results are presented in Table 3.

**Table 3:** Dimensions’ characteristics of Brief COPE

Dimension (Items)	*N*	M (SD)^a^	IIC	IDV	% Floor effect	% Ceiling effect	Alpha	INFIT
			min - max	min - max				min-max
Social support (8)	385	3.89 (1.27)	0.57 – 0.79	− 0.11 – 0.33	7.5	0.3	0.82	0.64 – 1.58
Problem solving (4)	391	4.57 (1.66)	0.72 – 0.78	0.20 – 0.37	10.0	3.3	0.74	0.91 – 1.15
Avoidance (10)	363	3.03 (0.75)	0.33 – 0.61	− 0.11 – 0.24	9.1	0.3	0.64	0.89 – 1.22
Positive thinking (6)	398	4.59 (1.23)	0.50 – 0.70	− 0.14 – 0.46	2.8	0.5	0.71	0.88 – 1.26

*M* (*SD*) mean (standard deviation); *IIC* item internal consistency; *IDV* item discriminant validity; Alpha Cronbach’s alpha; *INFIT* Rasch statistics

^a^scores ranging from 0 to 10; Higher scores reflect a higher tendency to implement the corresponding coping strategies

### External validity

Age was not linked to the coping strategies used by the individuals. Women more frequently used passive coping strategies, such as social support and avoidance, and less frequently used a positive thinking strategy compared to men. Individuals who were partners used social support coping strategies less frequently than the individuals who were not partners. Being single was more linked to avoidance strategies. Individuals with employment and individuals with a higher educational level frequently used problems solving strategies.

In general, the use of passive coping strategies, such as social support and avoidance, were negatively correlated with QoL, whereas the use of active coping strategies, such as problem solving and positive thinking, were positively linked with QoL. Mental health, vitality dimensions and the mental composite score of the SF36 were all linked to the coping strategies, whereas the physical composite score was not related to strategies used by the individuals. All the details are provided in Table 4.

**Table 4:** Relationships between Brief COPE and sociodemographics and quality of life

		Brief COPE			
		Social support	Problem solving	Avoidance	Positive thinking
Age		−0.009	−0.044	−0.001	−0.072
Gender	Women M (SD)	4.12 (1.22)	4.57 (1.59)	3.12 (0.70)	4.44 (1.65)
	Men M (SD)	3.62 (1.29)	4.60 (1.74)	2.92 (0.81)	4.77 (1.29)
	*p*-value	**<0.001**	0.844	**0.014**	**0.009**
Patient-caregiver relationship	Partner M (SD)	3.71 (1.25)	4.58 (1.66)	2.99 (0.76)	4.50 (1.25)
	Not partner M (SD)	4.19 (1.22)	4.48 (1.63)	3.12 (0.72)	4.60 (1.21)
	*p*-value	**<0.001**	0.549	0.115	0.427
Living	In couple M (SD)	3.84 (1.23)	4.61 (1.65)	2.98 (0.75)	4.53 (1.24)
	Single M (SD)	4.02 (1.36)	4.52 (1.67)	3.18 (0.74)	4.73 (1.21)
	*p*-value	0.200	0.636	**0.024**	0.156
Employment status	Workers M (SD)	4.11 (1.20)	4.91 (1.61)	2.96 (0.71)	4.65 (1.14)
	Not workers M (SD)	3.82 (1.28)	4.47 (1.66)	3.06 (0.76)	4.57 (1.26)
	*p*-value	0.064	**0.026**	0.303	0.603
Educational level	<12 y M (SD)	3.76 (1.21)	4.27 (1.56)	3.09 (0.76)	4.54 (1.34)
	> = 12 y M (SD)	3.99 (1.29)	4.89 (1.69)	2.98 (0.75)	4.66 (1.11)
	*p*-value	0.076	**<0.001**	0.145	0.348
SF-36	Physical function.	−0.052	0.103*	−0.144**	0.110*
	Social functioning	−0.086	0.061	−0.162**	0.316***
	Role physical	−0.086	0.017	−0.152**	0.020
	Role emotional	−0.123*	0.074	−0.170***	0.162***
	Mental health	−0.207***	0.195***	−0.319***	0.479***
	Vitality	−0.105*	0.220***	−0.181***	0.355***
	Bodily pain	−0.108*	0.087	−0.157**	0.060
	General health	−0.032	0.176***	−0.181***	0.248***
	PCS	−0.016	0.069	−0.090	−0.010
	MCS	−0.154**	0.151**	−0.244***	0.421***

M (SD) mean (standard deviation)

Brief COPE range [0–5]; higher score indicated higher use of coping strategy;

PCS physical composite score, MCS mental composite score; range [0–100]; higher score indicated higher QoL; bold values: *p* <0.05

*p < 0.050, **p < 0.010, ***p < 0.001

### Replication on patient and caregiver subsamples

**Fig. 1:**
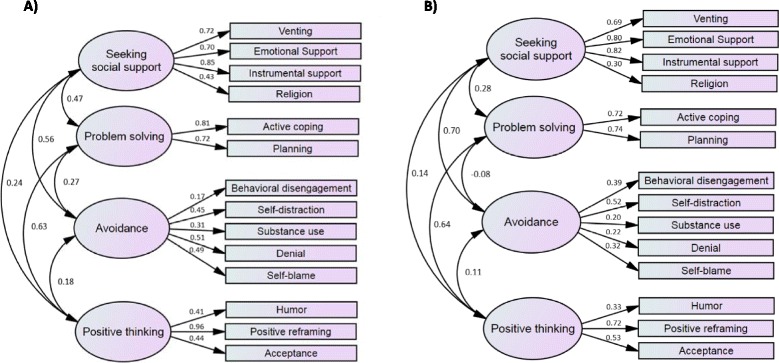
Factorial structure of Brief COPE for patients and caregivers. **a** Patients **b** Caregivers

The final 4-factor Brief COPE model showed a good fit on the two subsamples, patients and caregivers. All indices from the confirmatory LISREL model were satisfactory, with RMSEA = 0.047 and CFI = 0.923 for the patient subsample, and RMSEA = 0.031 and CFI = 0.938 for the caregiver subsample. The factorial structures of Brief COPE for patients and caregivers are presented in Fig. 1.

For the two subsamples, internal consistency was satisfactory for all dimensions. Each item achieved the 0.40 standard for item-internal consistency, except for the avoidance factor. The correlation of each item with its contributive dimension was higher than with the others, except for avoidance in the patient subsample. Cronbach’s alpha coefficients are satisfactory, except for the avoidance factor in the caregiver subsample. The scalability was satisfactory, except the social support factor. All results are presented in Additional file 1: Supplementary Table S2 and Supplementary Table S3.

## Discussion

The French version of the Brief COPE was validated in a large sample of students [11], and, in the authors’ opinion, showed satisfactory psychometric properties. The structure of the instrument was not modified, and use of the tool requires the assessment of 14 different scores. This study proposes, for the first time, a new structure of the French version of the Brief COPE, validated in a sample of individuals facing a singular stressful event, including cancer patients and their caregivers, with a smaller number of factors (four), which makes the instrument easier to use both in clinical practice and clinical research. Indeed, 4 indicators rather than 14 may be more practical and used to easily convey messages. Although the Brief COPE is an instrument that has been widely used in coping research worldwide, some publications previously performed surveys using different structures [9, 22] than the original Brief COPE version [7]. The authors provided structures with a different number of factors (eight [9], seven [22], five [23], and three [24–26] factors), explored in different sociocultural contexts [23, 26], and among various groups of individuals (healthy individuals, ill individuals [22, 25], and caregivers [23, 26]).

The present 4-factor structure is supported by different theoretical models of coping. First, for the model developed by Lazarus and Folkman that distinguishes problem-focused and emotion-focused coping strategies, we should consider that problem-focused strategies may be apprehended by the factor that we called ‘problem solving’ and the emotion-focused strategies by the dimensions we called ‘avoidance’ and ‘positive thinking’. The last dimension isolated by the factor analysis, named ‘social support’, remains controversial. According to authors, social support may be related to an emotion-focused strategy [2], a problem-focused strategy [27], and sometimes, it may be defined as an external social resource that an individual can benefit from and not a full-fledged coping strategy [28]. Suls and Fletcher proposed a closer model distinguishing avoidant and vigilant coping [29], a model that extensively overlaps the Lazarus’ model. A third theoretical model distinguished active (pacing and control) and passive (avoidance and support) strategies [4, 30]. In this last case, ‘avoidance’ and ‘social support’ may be considered passive strategies, whereas ‘positive thinking’ and ‘problem solving’ are active strategies.

This new 4-factor structure of the Brief COPE showed satisfactory psychometric properties. The structure presented acceptable internal consistency with all Cronbach’s alpha greater than 0.6. The items of one specific dimension were more correlated with their contributive dimensions than others. Some items had INFIT statistics outside the range [0.7–1.2]. This range is applicable in for the development of a new test, but is larger for an existing test, ranging from 0.5 to 1.5 [31]. Contrary to previous studies [32–34], age was not related to the strategies used by the individuals. Consistent with the literature [35–39], women more often used ‘social support’, including spirituality and religion, and emotional-focused strategies, including ‘avoidance’ and ‘positive thinking’, than men. Previous studies showed that the educational level and professional status might be associated with some specific coping strategies [40–42]. Associations between coping strategies and quality of life were elsewhere documented in other pathologic contexts [33, 43]. We found positive correlations between the positive thinking coping strategy and QoL dimensions, physical-like dimensions but particularly mental-like dimensions. It was previously demonstrated that positive emotions contribute to psychological and physical well-being [44, 45]. Similarly, the use of problem solving coping strategies seems to improve the QoL of the individuals. Problem-solving therapy interventions for patients and their caregivers showed positive findings on the QoL of individuals [46, 47]. Consistent with our findings, studies showed a negative correlation between avoidance and social support strategies and mental-like dimensions, social-like dimensions, [43], and physical-like dimensions of the QoL [48]. However, regarding the use of the social support coping strategy, particularly spirituality, some studies showed contradictory findings [49, 50], such that it may be associated with the improvement of well-being and social health. These discrepancies may be explained by the socio-cultural context and the greater or lesser part of spirituality within the social support dimension.

Some points should be discussed.

We preferentially attached some items to one of the 4 factors while the factor loading was less than 0.3, in accordance with the respective meanings of the item and the factor.

Some aspects of the validation process were not available at the time of this study, notably reproducibility, defined as the ability to produce the same results in the absence of a meaningful change. This property is the core psychometric property of a measuring instrument [51]. Assuming that what we are measuring isn’t changing, the measure would give us the same result over and over again. An unreproducible tool may compromise the validity of the measure, the precision of the measure, and consequently the use of the measure. However, examination of reproducibility requires longitudinal data collection. Future studies should explore these issues. Finally, assessment of external validity should be more extensive, and an exploration of links between the Brief COPE scores and burden, self-esteem, and perceived stress is lacking.

Authors previously explored the structure of the French version of the Brief COPE showing that the instrument could be represented with a 5-factor structure, but this publication did not provide extensive indicators that may prevent the use of this structure [52]. We believe that our present extensive work, including a more complete validation procedure, will provide potential users arguments supporting the relevance of the 4-factor structure.

## Additional file

Additional file 1: Replication on patient and caregiver subsamples. **Table S1**. Characteristics of patients and caregivers. **Table S2**. Dimensions’ characteristics of Brief COPE for patients and caregivers. **Table S3**. Relationships between Brief COPE and sociodemographics and quality of life for patients and caregivers. (DOCX 53 kb)

## Abbreviations

BP: Bodily Pain
Brief COPE: Brief Coping Orientation to Problems Experienced
CFA: Confirmatory factor analysis
CFI: Comparative fit index
GH: General Health
INFIT: Inlier-sensitive fit
KMO: Kaiser-Meyer-Olkin
MH: Mental Health
PF: Physical Functioning
QoL: Quality of life
RE: Role-Emotional
RMSEA: Root mean square error of approximation
RP: Role-Physical
SF: Social Functioning
SF36-MCS: SF36 Mental Composite Score
SF36-PCS: SF36 Physical Composite Score
VIT: Vitality
