# Supplementary material for: Assessment of coping: a new french four-factor structure of the brief COPE inventory
Source: Health Qual Life Outcomes. 2017 Jan 11;15:8. doi: 10.1186/s12955-016-0581-9 (PMC5225566; doi:10.1186/s12955-016-0581-9)
Supplement: Additional file 1: — Replication on patient and caregiver subsamples. Table S1. Characteristics of patients and caregivers. Table S2. Dimensions’ characteristics of Brief COPE for patients and caregivers. Table S3. Relationships between Brief COPE and sociodemographics and quality of life for patients and caregivers. (DOCX 53 kb) [file 12955_2016_581_MOESM1_ESM.docx]

**Supplementary table 1. Characteristics of patients and caregivers**

|  |  | **Patients** |
| --- | --- | --- |
|  |  | **N=235** |
| Gender^3^ | Women | 102 (44) |
|  | Men | 132 (56) |
| Age (years) | Mean (SD)^1^ | 60.5 (12.0) |
| Living^3^ | With a partner | 155 (67) |
|  | Alone | 77 (33) |
| Educational level^3^ | <12 years | 122 (53) |
|  | >= 12 years | 108 (47) |
| Professional status^3^ | Workers | 38 (16) |
|  | Not workers | 195 (84) |
| Cancer location3 | Head and neck | 64 (27) |
|  | Lung | 58 (25) |
|  | Gynecologic | 45 (19) |
|  | Urologic | 23 (10) |
|  | Gastroenteric | 7 (3) |
|  | Others* | 38 (16) |
| Metastasis^3^ |  | 71 (33) |
| Disease duration (months) | Median [IQR]^2^ | 25.6 [16.8;42.4] |
| WHO PS^3^ | 0  1  2 | 139 (63)  73 (33)  8 (4) |
|  |  | **Caregivers** |
|  |  | **N=163** |
| Gender^3^ | Women | 119 (74) |
|  | Men | 43 (27) |
| Age (years) | Mean (SD)^1^ | 54.8 (14.6 |
| Living ^3^ | With a partner | 132 (82) |
|  | Alone | 30 (18) |
| Educational level^3^ | <12 years | 74 (46) |
|  | >= 12 years | 85 (54) |
| Professional status^3^ | Workers | 52 (32) |
|  | Not workers | 108 (68) |

* sarcoma, bone, thyroid

^1^ SD standard deviation, ^2^ IQR interquartile range, ^3^ relative frequency,

WHO PS World Health Organization Performance Status

**Supplementary table 2. Dimensions’ characteristics of Brief COPE for patients and caregivers**

| Dimension (Items) | N | Mean (SD)* | Missing values (%) | IIC  min – max | IDV  min – max | % Floor effect | % Ceiling effect | Alpha | INFIT  min-max |
| --- | --- | --- | --- | --- | --- | --- | --- | --- | --- |
| 1. Patients |  |  |  |  |  |  |  |  |  |
| Social support (8) | 231 | 3.90 (1.33) | 13.16 | 0.57 - 0.81 | 0.13 – 0.36 | 9.09 | 0.43 | 0.83 | 0.64 – 1.53 |
| Problem solving (4) | 231 | 4.51 (1.70) | 10.12 | 0.74 – 0.76 | 0.14 – 0.41 | 12.55 | 3.03 | 0.73 | 0.94 – 1.18 |
| Avoidance (10) | 216 | 3.08 (0.81) | 17.87 | 0.31 – 0.64 | 0.15 - 0.33 | 11.11 | 0.46 | 0.67 | 0.83 – 1.24 |
| Positive thinking (6) | 235 | 4.66 (1.33) | 10.19 | 0.51 – 0.71 | -0.15 – 0.51 | 3.83 | 0.85 | 0.74 | 0.88 – 1.20 |
| 2. Caregivers |  |  |  |  |  |  |  |  |  |
| Social support (8) | 154 | 3.87 (1.19) | 18.52 | 0.57 – 0.77 | 0.17 – 0.35 | 5.19 | 0.65 | 0.81 | 0.65 – 1.68 |
| Problem solving (4) | 160 | 4.67 (1.61) | 10.61 | 0.69 – 0.79 | 0.17 – 0.32 | 6.25 | 3.75 | 0.75 | 0.85 – 1.06 |
| Avoidance (10) | 147 | 2.97 (0.66) | 23.04 | 0.35 – 0.58 | 0.18 – 0.30 | 6.12 | 0.68 | 0.58 | 0.85 – 1.24 |
| Positive thinking (6) | 163 | 4.48 (1.07) | 14.66 | 0.46 – 0.71 | 0.18 – 0.38 | 1.23 | 0.61 | 0.67 | 0.70 – 1.34 |

M (SD) mean (standard deviation); ICC item internal consistency; IDV item discriminant validity; Alpha Cronbach’s alpha; INFIT Rasch statistics

* scores ranging from 0 to 5; higher score indicated higher use of coping strategy

**Supplementary table 3. Relationships between Brief COPE and sociodemographics and quality of life for patients and caregivers**

|  | Patients |  |  |  |  |  | Caregivers |  |  |  |
| --- | --- | --- | --- | --- | --- | --- | --- | --- | --- | --- |
|  | Brief COPE |  |  |  |  |  | Brief COPE |  |  |  |
|  | Social support | Problem solving | Avoidance | Positive thinking |  |  | Social support | Problem solving | Avoidance | Positive thinking |
| Age | 0.038 | -0.063 | -0.077 | -0.140* |  | Age | -0.096 | 0.001 | 0.078 | -0.016 |
| Gender |  |  |  |  |  | Gender |  |  |  |  |
| Females M (SD) | 4.20 (1.23) | 4.51 (1.62) | 3.20 (0.71) | 4.50 (1.30) |  | Females M (SD) | 4.04 (1.21) | 4.62 (1.56) | 3.04 (0.68) | 4.40 (1.04) |
| Males M (SD) | 3.68 (1.37) | 4.52 (1.75) | 2.96 (0.87) | 4.80 (1.34) |  | Males M (SD) | 3.47 (1.05) | 4.84 (1.71) | 2.80 (0.57) | 4.70 (1.13) |
| p-value | 0.003 | 0.982 | 0.031 | 0.088 |  | p-value | 0.006 | 0.433 | 0.052 | 0.119 |
| Relationships |  |  |  |  |  | Relationships |  |  |  |  |
| Partner M (SD) | 3.64 (1.28) | 4.48 (1.70) | 2.98 (0.83) | 4.67 (1.33) |  | Partner M (SD) | 3.81 (1.21) | 4.73 (1.61) | 2.99 (0.67) | 4.51 (1.02) |
| Others M (SD) | 4.33 (1.26) | 4.38 (1.66) | 3.24 (0.76) | 4.56 (1.31) |  | Others M (SD) | 3.99 (1.15) | 4.61 (1.60) | 2.94 (0.64) | 4.43 (1.16) |
| p-value | 0.000 | 0.682 | 0.026 | 0.524 |  | p-value | 0.349 | 0.661 | 0.644 | 0.625 |
| Living |  |  |  |  |  | Living |  |  |  |  |
| In couple M (SD) | 3.79 (1.27) | 4.49 (1.70) | 3.00 (0.84) | 4.59 (1.38) |  | In couple M (SD) | 3.89 (1.20) | 4.74 (1.58) | 2.96 (0.62) | 4.46 (1.05) |
| Single M (SD) | 4.13 (1.43) | 4.53 (1.68) | 3.24 (0.72) | 4.80 (1.23) |  | Single M (SD) | 3.70 (1.08) | 4.50 (1.68) | 3.04 (0.78) | 4.54 (1.16) |
| p-value | 0.064 | 0.880 | 0.044 | 0.250 |  | p-value | 0.447 | 0.429 | 0.564 | 0.714 |
| Employment status |  |  |  |  |  | Employment status |  |  |  |  |
| Workers M (SD) | 4.31 (1.31) | 5.19 (1.60) | 3.05 (0.81) | 4.82 (1.11) |  | Workers M (SD) | 3.96 (1.11) | 4.73 (1.61) | 2.90 (0.62) | 4.52 (1.15) |
| Not workers M (SD) | 3.83 (1.32) | 4.38 (1.68) | 3.08 (0.81) | 4.63 (1.37) |  | Not workers M (SD) | 3.80 (1.19) | 4.66 (1.62) | 3.01 (0.67) | 4.47 (1.04) |
| p-value | 0.047 | 0.007 | 0.823 | 0.408 |  | p-value | 0.429 | 0.795 | 0.320 | 0.771 |
| Educational level |  |  |  |  |  | Educational level |  |  |  |  |
| >=12 y M (SD) | 4.10 (1.38) | 4.93 (1.77) | 3.10 (0.84) | 4.73 (1.22) |  | >=12 y M (SD) | 3.85 (1.16) | 4.85 (1.60) | 2.81 (0.56) | 4.56 (0.94) |
| < 12 y M (SD) | 3.73 (1.25) | 4.16 (1.54) | 3.05 (0.79) | 4.62 (1.41) |  | < 12 y M (SD) | 3.82 (1.14) | 4.45 (1.59) | 3.16 (0.71) | 4.41 (1.22) |
| p-value | 0.036 | 0.001 | 0.657 | 0.515 |  | p-value | 0.846 | 0.132 | 0.001 | 0.387 |
| SF-36 |  |  |  |  |  | SF-36 |  |  |  |  |
| PF | -0.042 | 0.171* | -0.085 | 0.213*** |  | PF | -0.068 | -0.052 | -0.223** | 0.004 |
| SF | -0.078 | 0.123 | -0.136* | 0.322*** |  | SF | -0.100 | -0.051 | -0.210* | 0.318*** |
| RP | -0.046 | 0.078 | -0.108 | 0.092 |  | RP | -0.157 | -0.119 | -0.175* | 0.024 |
| RE | -0.118 | 0.144* | -0.128 | 0.154* |  | RE | -0.127 | -0.043 | -0.218** | 0.227** |
| MH | -0.236*** | 0.232*** | -0.311*** | 0.482*** |  | MH | -0.166* | 0.161* | -0.390*** | 0.464*** |
| Vi | -0.051 | 0.296*** | -0.103 | 0.378*** |  | Vi | -0.200* | 0.076 | -0.316*** | 0.367*** |
| BP | -0.135* | 0.143* | -0.119 | 0.098 |  | BP | -0.062 | -0.025 | -0.188* | 0.063 |
| GH | -0.025 | 0.216*** | -0.158* | 0.297*** |  | GH | -0.029 | 0.076 | -0.188* | 0.303*** |
| PCS | 0.015 | 0.175* | -0.033 | 0.098 |  | PCS | -0.016 | -0.125 | -0.96 | -0.076 |
| MCS | -0.160* | 0.206** | -0.225*** | 0.422*** |  | MCS | -0.154 | 0.089 | -0.317*** | 0.418*** |
| Metastasis |  |  |  |  |  |  |  |  |  |  |
| Yes M (SD) | 2.99 (1.88) | 5.63 (1.82) | 2.04 (1.39) | 4.35 (2.01) |  |  |  |  |  |  |
| No M (SD) | 3.23 (2.36) | 5.84 (2.20) | 1.75 (1.38) | 4.42 (2.31) |  |  |  |  |  |  |
| p-value | 0.456 | 0.482 | 0.199 | 0.815 |  |  |  |  |  |  |
| Disease duration | 0.109 | -0.013 | 0.049 | 0.088 |  |  |  |  |  |  |
| WHO PS |  |  |  |  |  |  |  |  |  |  |
| 0 M (SD) | 3.36 (2.16) | 5.84 (2.01) | 1.84 (1.35) | 4.48 (2.29) |  |  |  |  |  |  |
| 1-2 M (SD) | 2.83 (2.27) | 5.58 (2.19) | 1.85 (1.37) | 4.21 (2.12) |  |  |  |  |  |  |
| p-value | 0.104 | 0.405 | 0.970 | 0.402 |  |  |  |  |  |  |

M (SD) mean (standard deviation)

Brief COPE range [0-5]; higher score indicated higher use of coping strategy;

PF Physical function. SF Social functioning. RP Role physical. RE Role emotional. MH Mental health. Vi Vitality. BP Bodily pain. GH General health. PCS physical composite score. MCS mental composite score; range [0-100]; higher score indicated higher QoL;

WHO PS World Health Organization Performance Status

*p<0.050. **p<0.010. ***p<0.001
